# A software solution for recording circadian oscillator features in time-lapse live cell microscopy

**DOI:** 10.1186/1747-1028-5-17

**Published:** 2010-07-06

**Authors:** Daniel Sage, Michael Unser, Patrick Salmon, Charna Dibner

**Affiliations:** 1 Biomedical Imaging Group, Ecole Polytechnique Fédérale de Lausanne (EPFL), Lausanne, Switzerland; 2 Department of Neurosciences, Faculty of Medicine, University of Geneva, Geneva, Switzerland; 3 Division of Endocrinology, Diabetes and Nutrition, University Hospital of Geneva (HUG), Geneva, Switzerland

## Abstract

**Background:**

Fluorescent and bioluminescent time-lapse microscopy approaches have been successfully used to investigate molecular mechanisms underlying the mammalian circadian oscillator at the single cell level. However, most of the available software and common methods based on intensity-threshold segmentation and frame-to-frame tracking are not applicable in these experiments. This is due to cell movement and dramatic changes in the fluorescent/bioluminescent reporter protein during the circadian cycle, with the lowest expression level very close to the background intensity. At present, the standard approach to analyze data sets obtained from time lapse microscopy is either manual tracking or application of generic image-processing software/dedicated tracking software. To our knowledge, these existing software solutions for manual and automatic tracking have strong limitations in tracking individual cells if their plane shifts.

**Results:**

In an attempt to improve existing methodology of time-lapse tracking of a large number of moving cells, we have developed a semi-automatic software package. It extracts the trajectory of the cells by tracking theirs displacements, makes the delineation of cell nucleus or whole cell, and finally yields measurements of various features, like reporter protein expression level or cell displacement. As an example, we present here single cell circadian pattern and motility analysis of *NIH3T3 *mouse fibroblasts expressing a fluorescent circadian reporter protein. Using Circadian Gene Express plugin, we performed fast and nonbiased analysis of large fluorescent time lapse microscopy datasets.

**Conclusions:**

Our software solution, Circadian Gene Express (CGE), is easy to use and allows precise and semi-automatic tracking of moving cells over longer period of time. In spite of significant circadian variations in protein expression with extremely low expression levels at the valley phase, CGE allows accurate and efficient recording of large number of cell parameters, including level of reporter protein expression, velocity, direction of movement, and others. CGE proves to be useful for the analysis of widefield fluorescent microscopy datasets, as well as for bioluminescence imaging. Moreover, it might be easily adaptable for confocal image analysis by manually choosing one of the focal planes of each z-stack of the various time points of a time series.

**Availability:**

CGE is a Java plugin for ImageJ; it is freely available at: http://bigwww.epfl.ch/sage/soft/circadian/.

## Background

Circadian oscillators have been described in virtually all organisms from cyanobacteria to humans. The mammalian circadian timing system has a hierarchical structure in that a master pacemaker residing in the suprachiasmatic nucleus synchronizes slave oscillators existing in most body cells [1]. Moreover, circadian clocks are ticking in mammalian cultured cell lines, like *Rat1 *or *NIH3T3 *fibroblasts, and these clocks are self-sustained and cell-autonomous [2,3]. A negative transcription/translation feedback loop, comprising clock genes repressing their own transcription, was proposed as the universal operational principle for generating circadian rhythm. Posttranslational events, like protein phosphorylation or acetylation, contribute critically to rhythm generation [4,5].

Recent advances in time-lapse fluorescent imaging have allowed new insights into the mechanisms of circadian rhythms. Luciferase enzymes have been extensively used as reporters for numerous purposes in organisms as diverse as cyanobacteria, plants, fruit flies, and mice [6]. Bioluminescence and fluorescence time lapse microscopy approaches have been successfully used to investigate molecular mechanisms of the mammalian circadian oscillator at a single cell level, the cross talk between individual cell clocks, and the mechanisms of single cell clock synchronization [3,7]. Transgenic *NIH3T3 *cell lines stably expressing a short-lived nuclear yellow fluorescent protein (Venus) from circadian regulatory elements of the *Rev-erbα *locus (Rev-VNP), or luciferase protein driven by circadian *Bmal1 *promoter (Bmal1-luc), have been established and exploited to unravel different aspects of mammalian circadian clockwork machinery [3,4,8].

In spite of remarkable potential of the time lapse microscopy to address various questions of circadian biology, there is a very limited number of data analysis software available. Commercially available software Metamorph (Universal Imaging Corp), Imaris (Bitplane A.G.) and DiaTrack (Semasopht) incorporate modules to track objects and to measure intensity in a region of interest. However, the analysis of the reporter protein level in the described above time lapse microscopy datasets using these software requires a lot of manual interventions. Metamorph interrupts tracking in every valley of the circadian cycle; therefore the user has to manually complete the trace. This is mainly due to the high variation of intensity in the reporter protein level from one frame to another. Approaches based on intensity threshold or on template matching are not able to perform a correct tracking. In addition, a manual analysis is unreasonably time-consuming and subject to errors in observer judgment.

In an attempt to go beyond the tracking capability of conventional software, we tailored our approach towards tracking over longer periods of time. To achieve this, we had to employ advanced image-analysis methods to filter away reliance on a strongly changing fluorescent or bioluminescence reporter signal. We developed this new user-friendly image-analysis software for accurate tracking of individual cells in a living cell population. Tools presented here allow tracking and segmentation of the cells under the conditions of cyclic variations of intensities. The standard approach to track is to decompose the problem into two steps: 1) the segmentation phase which extracts the objects from the background in a frame; 2) the linking phase which tries to find the best match between objects from one frame to the next frame. This is the "frame-to-frame tracking" paradigm, taken by most commercial software and by the majority of the research community [9]. A nearest-neighbor approach fails quickly because the cells have similar appearance in the valley of the circadian cycle. This approach can not be applied for the study of circadian oscillator. The circadian reporter protein expression level oscillates dramatically over 24 hours, to the extent that at the lowest point it comes close to the background level, making it difficult to distinguish the reporter level from the background. To resolve this tracking problem, we propose a solution where there is no explicit detection of the object to track. We formalize the tracking as an optimal process solving the shortest-path problem with the dynamic programming algorithm [10]. Therefore, extracting the cell trajectory consists of finding a path in the spatio-temporal volume 2D + T by optimizing a cost function. Practically, the optimization minimizes error based on the intensity and on the displacement. This procedure which takes into account the past and the future of the particles is efficient for tracking dim particles. The program first tracks the center of gravity of a cell over the whole sequence of images. For the images that have a good enough resolution to precisely delineate the contour of the cells, we have incorporated a segmentation tool to our software based on the active rays' methods. The tool allows contouring of the convex cell shape based on knowledge of the center of the cell. Finally, the program makes measurements of several parameters like size, mean protein expression, displacement, etc. In addition, we have developed a granulometry application, which allows following of protein sub-nuclear localization during the cell cycle. Similarly to endogenous DNA ligase I, RFP-DNA ligase I construct is associated with the site of DNA replication (replication foci) during S phase. Thus RFP-DNA ligase I sub-nuclear distribution changes from punctuated (during S-phase) to dispersed, which makes it possible to easily follow cell cycle progression [11]. Our granulometry application discriminates between condensed and dispersed (homogenous) states of the protein, allowing in the case of RFP-DNA ligase I protein to obtain the accurate time frame for S-phase, and thus to follow cell cycle progression.

The tools are written as Java plugin of the popular ImageJ image-processing software package (ImageJ: National Institutes of Health, Bethesda, MD, USA). Our experimental results show that this semi-automatic analysis method is reliable, reproducible and efficient for individual moving cell tracking, fluorescence or bioluminescent protein expression level quantification and cell trajectory analysis.

## Results

### Algorithm

#### 1. Tracking of cells with highly variable intensities

The task of automated tracking of moving objects has been studied extensively in the digital image analysis literature. In biology, tracking is of fundamental importance in cell motility studies. Different techniques have been proposed in the past for tracking cells or particles in the context of biological imaging [12]. However, the common tracking paradigm, which consists of pre-processing the image, detecting the objects, and linking them from frame to frame, fails in the application to circadian expressed proteins. Due to extreme variations in reporter protein expression level over 24 hours, there is a high risk of loosing the tracked cell at the lowest phase. At this point the grey-level intensity (corresponding to the level of protein expression) often approaches the background value.

For our application, the following observations should be taken into account in the design of a specific tracking algorithm:

(1) The cells are approximately round.

(2) Each cell leaves a unique trace; in the case of cell division, the requirement is to track a single one of the daughter cells. The circadian rhythm study does not need to recover cell division lineage.

(3) The intensity of a cell can approach that of the background for short periods of time.

**Figure 1:**
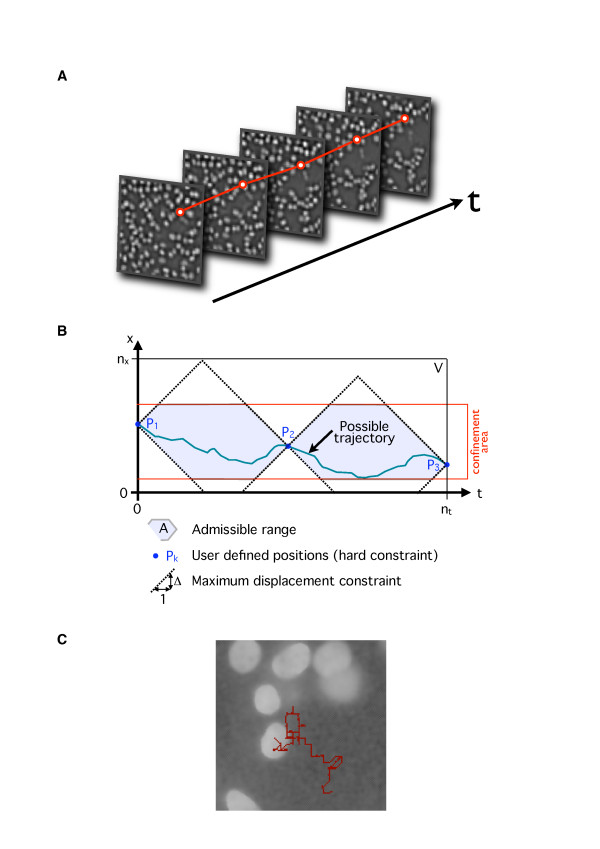
**Tracking cell using DP**. **A**. 5 frames extracted from an image sequence showing the results of the preprocessing step (Laplacian of Gaussian filter) and the reduced version of the images. Cells appear as particles consisting of a few pixels. The red line represents an example of a trajectory. **B**. Schematic representation of the admissible range for the dynamic programming (DP) algorithm. An example of a possible trajectory inside the admissible range is shown in blue. In this simplified diagram only one of the spatial dimensions (X) is shown; in the real implementation there exist two spatial dimensions X and Y, which define the admissible range as a volume. The slope of the dotted boundaries of the admissible range corresponds to the maximum velocity of the particles computed from the maximum displacement value Δ. **C**. Example of a cell trajectory overlaid over the first frame of the sequence. The trajectory is staircase due to reduction of image.

With these assumptions, we formulated the tracking process as a shortest-path problem which admits an optimal solution. This optimal path is a spatio-temporal trajectory consisting of a set of ordered time-position {(**x**_1_), {(**x**_2_),...,(**x**_n_)} for t = 1 to n, where the discrete state **x**_t _is a vector of coordinate (x, y) and *n *is the number of frames shown in Fig. 1A. The trajectory is hence extracted from the spatio-temporal (2D + T) volume V. A solution to the optimal path problem in this context (discrete ordered state space) is afforded by the dynamic programming (DP) algorithm which is an optimization procedure searching for the optimum of a cost function. DP was already used for the purpose of tracking fluorescent particles in noisy images [10]. DP was used to track the gravity centers of individual cells. The original image f(**x**) was first preprocessed using a Laplacian of Gaussian filter given Δf(**x**). This filter has the desirable properties of suppressing background noise and enhancing Gaussian-shaped objects.

Due to the combinatorial explosion in the number of possible trajectories, the DP algorithm can quickly become extremely expensive in terms of computational cost. To overcome this problem we impose several severe constraints in forming the search graph:

(1) Images are down sized so that single cells are reduced to particles of a few pixels. Size reduction provides a significant gain in terms of computational cost but, obviously, one loses in spatial precision. However, at this stage, a rough estimate of cell positions is sufficient.

(2) Choice of start and an end point. DP is performed between user-specified start and end positions P_1 _(**x**_1_) and P_n _(**x**_n_). The user can interactively choose the cell to track and provide the necessary positions.

(3) Introduction of a confinement area. It is assumed that the cell cannot exit the area between P_1 _and P_N _with a margin.

(4) Maximum displacement constraint. It is additionally assumed that the cell displacement between consequent frames cannot exceed a certain step size Δ.

These constraints define the admissible range A as a subset of the original volume V as shown in Fig. 1B. The DP algorithm first evaluates the cost (score) of all possible trajectories by an exhaustive search initiated from a given starting point within the admissible range. The optimal solution is then determined by means of a backtracking procedure. In our implementation, we defined the following cost function between two positions **u**(x_u_, y_u_) and **v**(x_v_, y_v_):

This cost function (which is to be minimized) includes two standard terms known as external and internal energies in snake terminology. The external energy term depends on the data and favors small variations of intensities, while the internal energy is a spatial regularization term that penalizes large variations of displacements. We additionally included a third term favoring intensities close to the intensity of the starting or end points. The three coefficients λ_ext_, λ_int_, and λ_fix _are weighting parameters that also serve as normalization factors. They can be tuned by the user to match the requirements of specific problems. The internal energy term is particularly important in the tracking of particles with low observability; it allows the algorithm to pursue the tracking according to a smooth-trajectory assumption, even when the cells in question have weak protein expressions.

DP is a highly efficient and robust algorithm for extracting spatio-temporal trajectories of particles (Fig. 1C), even in difficult cases where the particles have low intensities. The method is, however, adequate only for tracking individual particles, and does not straightforwardly extend to the joint tracking of several particles. For multiple-trajectory analysis, the user has to choose the cell to track, run the tracking process, and repeat the same operations for other cells.

#### 2. Cell shape delineation

**Figure 2:**
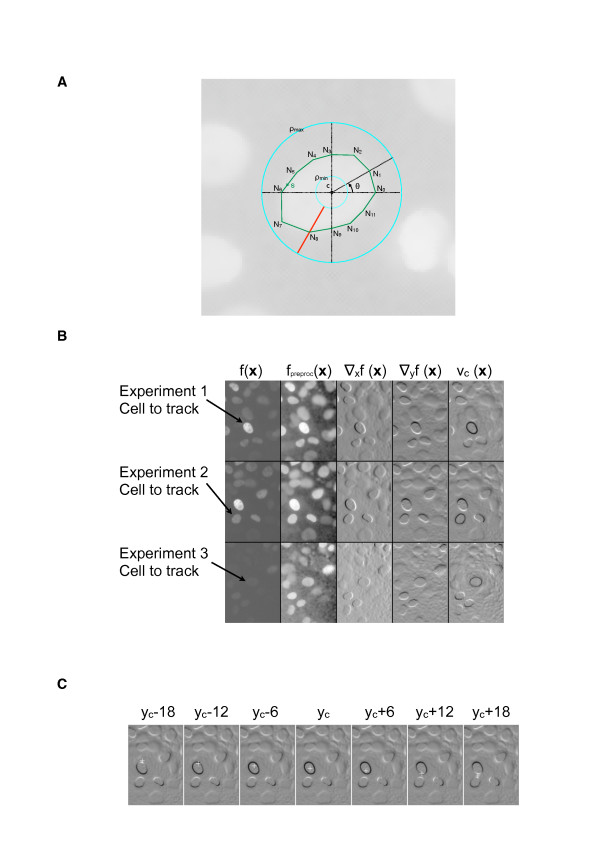
**Segmentation of cell**. **A**. Parametrization of the active rays. Illustration of a contour curve overlaid on a real cell image. In this example, the curve is constructed using 12 nodes that are interpolated linearly. The two concentric circles mark the limits of the search space for ρ. The center **c **is given by the tracking procedure. **B**. Illustrations of the consecutive steps for computing the potential function under three different experimental conditions. The original image f(**x**) is first preprocessed and rescaled f_preproc_(**x**); next, a gradient is computed in the X and Y directions, in order finally to compute the potential function ν_**c**_(**x**). **C**. Stability of the potential function ν_**c**_(**x**) with respect to variations in the position of the center **c**(x_c_, y_c_). In this illustration, the center was artificially shifted in the vertical direction and the potential function was recomputed. The shifted center is represented by a white cross. Shifts of -18, -12, -6, 0, 6, 12, and 18 pixels were considered. For translations of -18 and 18 pixels, the shifted center is placed outside the cell and the potential function does not express a clear black contour; on the contrary, for shift inferior to 12 pixels the potential function provides a clearly defined contour.

Once the approximate center of the cells has been found in every frame of the image sequence, their exact shapes can be identified using the computationally-efficient algorithm proposed below. The assumption underlying the method is that the shape is convex, which is the case for the studied cells in our applications. We defined the contour of a cell by *n *nodes specified by their polar coordinates (ρ, θ) given along rays R_k _originating at the known center **c **(x_c_, y_c_) as shown in Fig. 2A. This unique ordered parameterization makes it possible to formulate contour detection as a shortest-path problem, which, in turn, allows us to once again use dynamic programming (DP) to find this optimal path [13]. This method - known by the name "active rays"- was first introduced by Denzler [14]. The active rays method yields an optimization within a very limited discrete space, as both ρ and θ are sampled. The search space is defined by the center **c**, a minimum radius ρ_min_, a maximum radius ρ_max_, and the type of parameterization. The active rays' method offers two main advantages: the radial parameterization prevents intersections in the contour; and, the change of degrees of freedom per contour node to one parameter (ρ) reduces the computation cost substantially. However, one drawback of the method is its more limited accuracy compared to active contours as a result of discretization. The discretized radial parameterization does not permit the algorithm to follow fine circumvolutions of the contour and to extract concave contours. For the purpose of applying the active rays' method we formulated the following potential function:

where ∇f(**x**) is the gradient of an image f(**x**). This potential function depends on the position of the center c of the cell. We show in Fig. 2B that, as long as **c **remains inside the cell, the potential function suitably identifies the cell contour. The cost function to minimize consists of two terms:

The external term attracts the curve towards the contour via the potential function, while the internal energy term favors smooth curves. The final curve is then represented by a cubic spline that interpolates the nodes N_k_. In the implementation, images are preprocessed in order to reduce noise; Gaussian smoothing and a non-linear diffusion filter are applied to each frame in the sequence (Fig. 2B).

#### 3. Cell Feature Measurement

Quantities such as the level of gene expression as well as certain dynamic variables (velocity, direction of movement, and variations in the direction) are among interesting features that can be computed from the information provided by the tracking module, for both bioluminescent and fluorescent images. Other features, such as the size of the cells and mean levels and standard deviations of gene expression, are calculated by also taking into account the shape information obtained using the shape delineation module. Sudden changes in the cell size (from large to small) can be used to detect cell divisions.

For a typical sequence of 200 frames, the software is able to track one cell in less than 200 ms, and to extract the shape in less than 2 s (Apple MacPro DualCore 2.66 MHz). The user has to enter few (around 5) positions by clicking on the cells to track. All the measurements are reported in a spreadsheet format and in graphical form.

### Testing: Comparison of cell oscillation pattern analysis by CGE to the existing method

**Figure 3:**
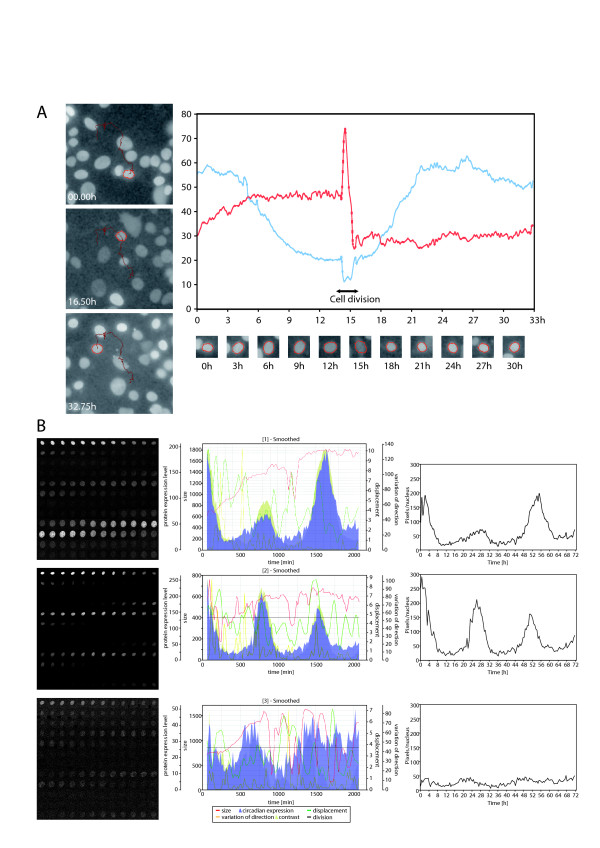
**Quantification of Rev-VNP circadian expression, cell displacement and nucleus size in *NIH3T3 *fibroblasts using CGE**. *NIH3T3 Rev-VNP *cells were synchronized using dexamethasone, and time-lapse microscopy of VNP fluorescence in the nuclei of individual cells was performed. Images were taken every 30 min for three consecutive days. The movies were created from the time-lapse series using *Leica AS MDW *software and analyzed using CGE *ImageJ *plug-in. **A**. Left panels: an individual nucleus tracked during 33 hours. Graph on the right: blue curve corresponds to Rev-VNP fluorescence level per nucleus; red curve reflects nucleus size over the time. **B**. Time lapse microscopy of Rev-VNP fluorescence in three individual nuclei over 72 hours. For each nucleus, the fluorescence intensity over the nucleus, nucleus size, nucleus displacement and variation of direction, were quantified and plotted against time. Time of cell division is marked when applicable (see **A.**).

**Figure 4:**
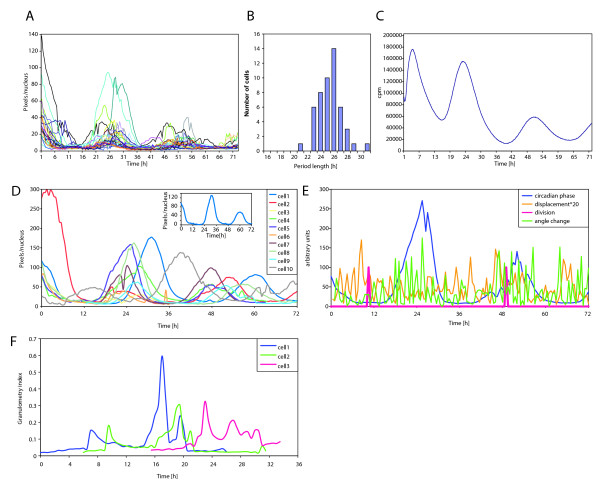
**Circadian fluorescence pattern and cell motility quantification in *NIH3T3 Rev-VNP *cells by CGE**. Time lapse microscopy movies with *NIH3T3 Rev-VNP *cells were performed and analyzed using CGE ImageJ plugin. **A**. Circadian fluorescence profiles of 50 individual cell nuclei. Period length was calculated as time span between two fluorescence peaks of individual cells after the dexamethasone shock: mean = 25.14 h, SD = 1.56 h, n = 50. **B**. Period length distribution. Normal distribution was observed, similar to Nagoshi et al., 2004. **C**. Circadian expression of *Reverba-luciferase *in *NIH3T3 *fibroblasts measured in the Actimetrics lumicycler. Period length from 3 experimental repeats was 25.45 h ± 1.82 h. **D**. Correlation of cell size and cell circadian phase/period length in *NIH3T3 Rev-VNP *fibroblasts transduced with LTAg SV40. **E**. Correlation of cell motility, cell division and cell circadian phase. **F**. Cell cycle analysis in *NIH3T3 *cells transiently expressing RFP-DNAI ligase.

Time lapse microscopy analysis of circadian Rev-VNP fluorescence in the nuclei of individual *NIH3T3 *fibroblasts (Additional file 1: Movie 1) using CGE is presented in Fig. 3. Following semi-automatic nucleus tracking (red line in the left panels in Fig. 3A), a number of output parameters could be obtained. Namely, circadian Rev-VNP intensity profiles (Fig. 3A, blue line in the graph; 3B middle and right panels), nucleus size (Fig. 3A, red line in the graph), nucleus displacement (the absolute distance and the variation of direction measured by trajectory angle change), and the cell division time (Fig. 3A) are outlined for the nuclei examples presented here. Of note, due to the preprocessing of the images by CGE, also the nucleus profile with relatively low circadian amplitude (the lowest panel in Fig. 3B) could be easily analyzed for circadian amplitude, phase, and period length. As a proof of principle, we compared the oscillation profiles of individual *NIH3T3 Rev-VNP *cells obtained by CGE, to those calculated using the MetaMorph software, as described in Nagoshi et al., 2004 [3]. Briefly, in the previous study the centers of the fluorescent regions (i.e., centers of the nuclei) were followed manually throughout the time lapse series, and the fluorescence intensities of thus defined individual nuclei were calculated. The region of interest (ROI) was defined to cover all the fluorescence in the same cell from the first to the last frames, and the fluorescence intensity in the given ROI was measured. The same experimental design and conditions of image acquisition were used in both cases. As shown in Fig. 4A and 4B, individual cell oscillation profiles, circadian phase and period length distribution are in a good agreement with data published by Nagoshi [3] (Fig. 1D, E), and with our own calculations made by the method developed and used by Nagoshi and coworkers (not shown). The difference in the average period length obtained by us using CGE (25.14 ± 1.56 hours) and obtained by Nagoshi et al. using MetaMorph manual analysis (28.2 ± 2.9 hours) is most probably attributed to the difference in serum (FCS) content in the medium (10% in our case vs 0.5% in Nagoshi et al.). In addition, our analysis of *NIH3T3 *cells transiently expressing luciferase driven from *Reverbα *circadian promoter at the population level revealed very similar circadian period and phase characteristics (Fig. 4C). Furthermore, the average circadian period length revealed by our analysis of 50 individual *NIH3T3 Rev-VNP *fibroblast profiles by CGE was very close to the period calculated for confluent *NIH3T3 Reverbα-luciferase *fibroblasts at the population level (25.45 ± 1.82 hours; compare Figure 4A to 4C). Thus, CGE allowed us to perform analysis of cell circadian protein expression level, oscillation phase and period length, and the results obtained are in good agreement with those calculated by different methods. In contrast to existing methods, CGE permitted semi-automatic analysis of large datasets in reasonable time with high accuracy.

### Implementation

#### 1. Measurement of cell motility, nucleus size and cell division timing

In addition to cell circadian oscillation profile, we assessed other output parameters of CGE which are useful for numerous purposes in cell biology, such as cell nucleus size, cell motility and cell division time. Natural variations in cell size are remarkable between wild type individual *NIH3T3 *fibroblasts. These variations seem to be even more pronounced in *NIH3T3 *cells transduced with Large T Antigen of SV40 virus (LTAgSV40; Additional file 2: Movie 2). We used CGE to analyze oscillation pattern in correlation with these cell nucleus size. As depicted in Figure 4D, single cell analysis of *NIH3T3 Rev-VNP LTAgSV40 *fibroblasts indicated that similarly to parental cell line, these cells exhibit strong circadian oscillation pattern. About 15% of transformed cells had unusually large nucleus size (nucleus square ~1500 pixels^2^; example cell 1 (1973 pixels^2^; Additional file 2: Movie 2). Interestingly, increase in the nucleus and cell size correlated in our example experiment with longer oscillation period length: 25.5 h ± 0.8 h in cells with nucleus size smaller then 600 pixels^2 ^(395 ± 90 pixels^2^, n = 9), in comparison to 27.5 h ± 1.3 h in cells with nucleus larger then 1500 pixels^2^. Circadian phase might also be delayed in larger cells (see cell 1 profile at the small graph in Figure 4D). Thus LTAgSV40 over expression did not change cell oscillation pattern in most of the cells, however it might affect circadian period length and circadian phase in the cells harboring larger size. Larger datasets have to be analyzed to confirm a possible correlation between cell size and cell circadian pattern.

Cell motility was evaluated using two parameters: the distance and the movement angle change (direction evaluation) between each two subsequent time points. Using a small subset of cells (n = 20), we measured cell circadian phase, cell motility and cell division time (as demonstrated in an example cell in Fig. 4E). In a good agreement with previous publications [15], cell motility was increased after the cell division. No significant correlation was found between cell circadian phase and cell motility.

Thus, in addition to cell circadian oscillation profile, our software gives accurate evaluation of cell nucleus size, cell motility (distance and angle) and cell division time. It allows semi-automatic tracking and reliable analysis of various parameters during long term experiments implying large data sets of the cells exhibiting highly varying fluorescent protein levels and changing significantly their position during the experiment.

#### 2. Single cell oscillation profile analysis using bioluminescence time lapse microscopy large data sets

Fluorescent time lapse studies expose cells to strong light excitation, and therefore may be prone to toxic effects when using shorter wavelengths over longer periods of time. In our hands treatment of mouse fibroblasts with different drugs in combination with cell excitation every 30 minutes biased the experimental analysis due to cell poor condition, and to significant cell death. Recently developed bioluminescent microscopy, absolutely non-toxic for the cells [7], might represent an ultimate solution for the toxicity problem in long term experiments. We have successfully applied CGE method in order to analyze large datasets obtained from bioluminescent time lapse microscopy. Using this approach, we have recently unraveled intriguing questions of transcription and temperature compensation of mammalian circadian oscillator [8]. Time lapse microscopy of *NIH3T3 Bmal1-luc *fibroblasts was performed in this study, and at least 130 cells were tracked with subsequent analysis of their oscillation profiles using CGE. Thus in addition to fluorescent time lapse microscopy analysis, CGE could be used to analyze data sets obtained by bioluminescent time lapse microscopy.

#### 3. Cell cycle analysis

An extra feature we have developed for CGE, granulometry, allows the accurate follow up of protein sub-nuclear localization, specifically the discrimination between condensed and diffused protein expression pattern. In order to evaluate S phase time frame, which corresponds to condensed expression phase of RFP-DNA Ligase I construct [11], time lapse microscopy of *NIH3T3 *cells transiently expressing RFP-DNA Ligase I was performed as described in Supplementary Methods. In examples presented at Additional file 3: Movie 3 and Figure 4F experiment, the cell number 1 enters S phase 15 hours after the start of recording, staying in S-phase about 6 hours, and proceeding to G2 phase 21 hours after the beginning recording. Cell 2 stays in S-phase between 16 and 22 hours, cell 3 - between 22 and 28 hours. In the future, we are planning to use this experimental system and CGE granulometry analysis to follow the cell cycle and circadian clock in the same cell.

## Discussion and Conclusions

In conclusion, the CGE package allows one to study accurately and efficiently the different features and changes of cells with significantly varying locations and protein expression levels imaged over a period of several days. Our newly developed software proves to be more reliable, reproducible and efficient for individual cell circadian pattern quantification, in comparison to results obtained by different methods (MetaMorph). It is suitable for analysis of oscillation pattern from large data sets obtained by both fluorescence and bioluminescence time lapse microscopy. Experimental results herein and from our previous publications suggest that CGE allows accurate evaluation of fluorescent or bioluminescent reporter protein level and easy correlation between cell oscillation pattern and cell size, cell motility pattern and cell division timing. The granulometry feature provides accurate evaluation of protein sub-nuclear distribution, useful for cell cycle progression analysis, and allowing easy correlation between cell clock and cell cycle by the same experimental approach. More generally, our method offers a wide range of possibilities for cell tracking, cell size and cell motility analyses, for acquiring the protein distribution pattern and evaluation of cell cycle progression, making it useful in various aspects of cell biology research.

## Methods

### Cell lines and constructs

*NIH3T3 *cell line stably expressing short-lived nuclear fluorescent protein Venus-NLS-Pest1 driven from circadian *Reverbα *regulatory sequences (*NIH3T3 RevVNP*) was established as described in Nagoshi et al. [3]. To obtain LargeT Antigen SV40 (LTAg SV40) expressing cells, *NIH3T3 Rev-VNP *fibroblasts were transduced with *LTAgSV40 *expressing lentiviral particles as described by P. Salmon and colleagues [16,17] with subsequent clonal line selection. Parental and transformed *NIH3T3 *cells were maintained in DMEM supplemented with 10% FCS.

### Fluorescence Time lapse Microscopy

For time lapse microscopy experiments, cells were plated in 35-mm glass bottom dishes (WillCo-dish, type 3522, WillCo Wells B.V.) and grown to confluence. After stimulating the cells with 100 nM dexamethasone for 30 minutes, the medium was replaced by 2 ml phenol red-free DMEM supplemented with 10% FCS. The cultures were placed in a 37°C chamber equilibrated with humidified air containing 5% CO_2 _throughout the microscopy. Time lapse microscopy was performed with a Leica AF6000LX microscope using a 20× objective. For the cell cycle follow up experiments, *NIH3T3 *cells were plated in 35-mm Falcon plastic dishes, transiently transfected with RFP-DNAI ligase [11], and microscopy was started 24 hours after transfection using 40× objective. Time lapse images were captured with either a Roper Coolsnap HQ or a Cascade B CCD camera using a YFP filter set (Additional file 1 and 2: Movie 1, 2) or RFP filter set (Additional file 3: Movie 3).

## Competing interests

The authors declare that they have no competing interests.

## Authors' contributions

DS wrote the algorithm and contributed significantly to the study design and the draft writing. MU contributed to the study design, algorithm writing and critical revision of the manuscript. PS carried out the cell transduction experiments and contributed to manuscript revision. CD participated in study design and coordination, performed most of the experiments and data analysis, and wrote the manuscript draft. All authors read and approved the final manuscript.

## Supplementary Material

Additional file 1 **Movie 1**. Time lapse microscopy of Reverbα-Venus-NLS-PEST1 (Rev-VNP) protein circadian expression in *NIH3T3 *cells. Click here for file

Additional file 2 **Movie 2**. Time lapse microscopy of Rev-VNP protein circadian expression in *NIH3T3 *cells transduced with LTAg SV40. The overlaid drawings (outline + trace) are displayed over frame-to-frame rescaled images. Click here for file

Additional file 3 **Movie 3**. Time lapse microscopy of RFP-DNAI ligase transient expression in *NIH3T3 *cells. The overlaid drawings (outline + trace) are displayed over frame-to-frame rescaled images. Click here for file
